# South American Nothochrysinae (Neuroptera, Chrysopidae): II. Redescription of *Leptochrysaprisca* Adams & Penny

**DOI:** 10.3897/zookeys.866.35396

**Published:** 2019-07-24

**Authors:** Catherine A. Tauber

**Affiliations:** 1 Department of Entomology, Comstock Hall, Cornell University, Ithaca, NY, 14853, USA Cornell University Ithaca United States of America; 2 Department of Entomology and Nematology, University of California, Davis, CA, 95616, USA University of California Davis United States of America

**Keywords:** Female abdomen and genitalia, Green lacewing, Limaiinae, parasitoid

## Abstract

*Leptochrysa* Adams & Penny is one of four genera of Nothochrysinae recorded from the New World. Previously, this genus and its only described species, *Leptochrysaprisca* Adams & Penny, were known from a single female specimen that is discolored and damaged by fungal infestation. Thus, accurate information on the taxon was limited mostly to the wings and some other external features. Here, I describe a recently collected, second female specimen with the goal of providing images of the adult coloration and elucidating characters (especially the female genitalia) that were unavailable earlier. Some variation between the two known specimens is also noted and used in interpreting venation characters. Finally, comparisons are made with other extant genera pertaining to the placement of the species within Chrysopidae.

## Introduction

This article is the second of two that focus on recently found specimens of South American Nothochrysinae (Neuroptera, Chrysopidae). The family Chrysopidae currently consists of four subfamilies: three with extant representatives (Apochrysinae, Chrysopinae, and Nothochrysinae) and a single subfamily (Limaiinae) known only from fossils (see Archibald and Makarkin 2015). Among the extant Nothochrysinae the monotypic genus *Leptochrysa* Adams & Penny is the most enigmatic. Its systematic relationships are not well resolved; indeed, its assignment to Nothochrysinae is seriously questioned (Makarkin and Archibald 2013 and Discussion below). Moreover, because of its rarity, it has not been included in any molecular analyses.

This genus is known from only one species, *Leptochrysaprisca* Adams & Penny, and also from only one specimen, the badly damaged female holotype collected in Amazonas, Peru. Because this specimen was infested with fungal mycelia, Adams and Penny (1992b) mainly described external features, with emphasis on wing characteristics. Fortunately, an additional specimen, another female, has become available for study; below it is described with emphasis on abdominal and genital characters, as well as the color pattern of the exoskeleton. To allow the genus to be compared with those in recent morphology-based phylogenetic studies of the Chrysopoidea (e.g., Makarkin and Archibald 2013, Breitkreuz 2018), an effort was made to record relevant characters used in those studies.

## Material and methods

As explained in the previous article on *Nothochrysa* McLachlan (Tauber 2019), I did not determine tracheal pathways in order to identify the various veins and cells of the wings. Here, I used the obvious pathways of the veins and the findings of previous authors (mainly Adams 1967, Makarkin and Archibald 2013, and Breitkreuz et al. 2017). The report uses prevailing terminology for veins and cells, as most recently modified by Breitkreuz et al. (2017), with exceptions and additions as described by Tauber (2019). The terminology for other body parts conforms to common usage. Measurements reported here are only from the undescribed specimen; some measurements of the holotype were reported by Adams and Penny (1992b).

### Leptochrysa prisca

Taxon classificationAnimaliaNeuropteraChrysopidae

Adams & Penny, 1992

29b096a6-8796-43f4-afc2-ed15a05341ae

Figs 1 , 2 , 3 , 4 , 5 , 6 , 7 , 8 , 9 , 10

#### Material studied.

A single female specimen was found during a visit to the Florida State Collection of Arthropods (FSCA). Subsequent searches by L. A. Stange did not yield additional examples. The labels (all white) on the specimen read: [1] “PERU: Amazonas Dept / Huembo Lodge, Km / 315 on N5, 18-21-X- / 2012, 2078 m, JE Eger”; [2] “05°51'28.1S / 077°59'04.8W / MV & UV Light”; [3] “*Leptochrysaprisca* / Adams & Penny, det. / C. A. Tauber 2019”.

The specimen is well preserved, and its wings are spread. After imaging, the abdomen was cleared for study; it is held in a microvial containing glycerin, attached to the pin. During clearing, several parasitoid larvae were discovered in the abdominal cavity. They were removed, imaged, and preserved in a separate genitalia vial with glycerin.

For comparison, I examined the *L.prisca* holotype, which also was collected in the Peruvian region of Amazonas. The type locality is: “PERU. DEPT. AMAZONAS: 18 km N of Puente Engenio, km 320, alt 1750 m, 9 Oct. 1964, P. C. Hutchinson & J. K. Wright, collected on *Baccharislatifolia* #6380”. As noted above, the abdomen of this specimen is in poor condition, and the body and wings are discolored by the intrusion of dark mycelia. However, the wings and external structure of the specimen are well preserved; the gut contents and cleared abdomen are held in separate vials in the unit tray holding the specimen.

#### Classification – subfamily.

**Figure 1.**
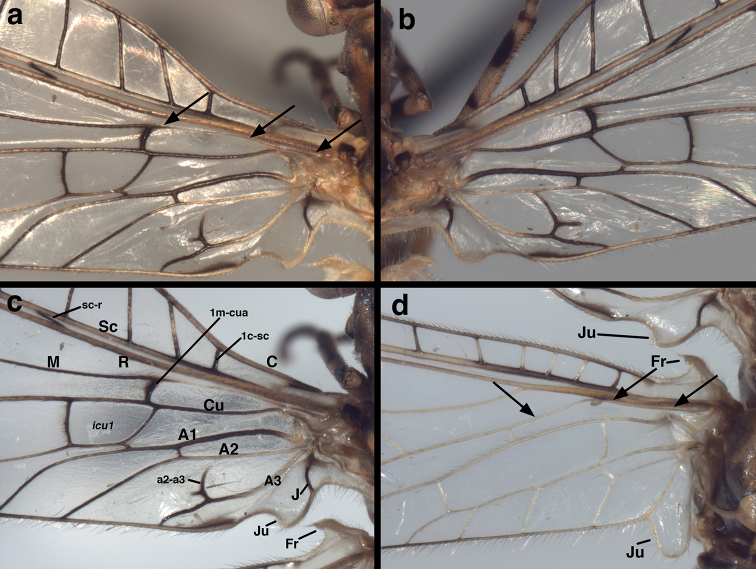
*Leptochrysaprisca* Adams & Penny (Peru, Amazonas, female, FSCA): Venation at base of wings (**a**) left forewing, (**b**) right forewing, (**c**) left forewing, labeled, (**d**) left hindwing, labeled. Note the absence of a tympanal organ at the base of R (forewing), the independent origin and trajectory of M along the base of R [forewing and hindwing; see arrows in (**a**) and (**d**), and the short break at the base of a2-a3 (forewing)]. Because of the natural pleating of the wings, the space below the Sc appears very small relative to its actual size. The sc-r crossvein is actually slanted as shown. **A1, A2, A3** first through third anal veins **a2–a3** crossvein between A2 and A3 **C** costa **Cu** cubitus **1c-sc** first crossvein between the costa and subcosta **Fr** frenulum ***icu1*** first intracubital cell **J** jugal vein (forewing only) **Ju** jugal lobe **M** media **1m-cua** first crossvein between the media and anterior cubital branch **R** radius **Sc** subcosta **sc-r** basal crossvein between the subcosta and radius.

**Figure 2.**
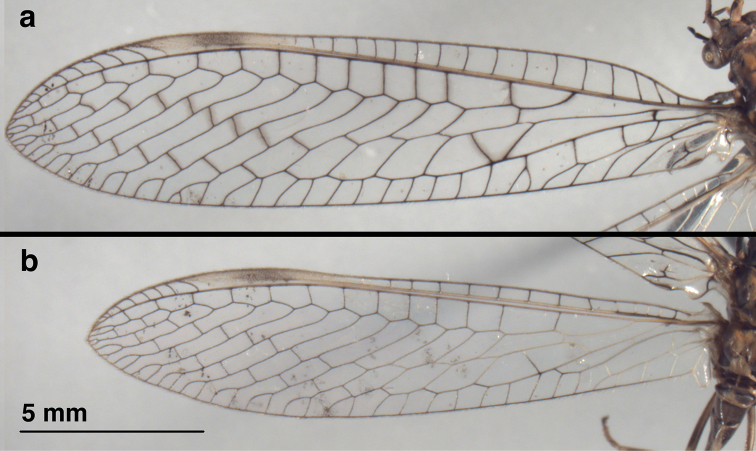
*Leptochrysaprisca* Adams & Penny (Peru, Amazonas, female, FSCA): Wings (**a**) forewing (**b**) hindwing.

The holotype and the specimen described here exhibit the following diagnostic features of adult Nothochrysinae (cf.: Tjeder 1966, as Dictyochrysinae; Adams 1967; Brooks and Barnard 1990; Makarkin and Archibald 2013; Breitkreuz 2018): (i) wing-coupling mechanism consisting of a large jugal lobe on the forewing and a frenulum on the hindwing (Figs 1, 2, 6); (ii) base of the forewing without tympanal organ (Fig. 1); (iii) forewing (and hindwing) with stem of the media (M) extending basally adjacent to the radius (R), not fused with it (see Fig. 1; cf. Breitkreuz 2018: 640); (iv) pseudomedia (Psm) merging (or appearing to merge) with inner gradates (not outer gradates) (Figs 2, 6); (v) pseudocubitus (Psc) merging with outer series of gradates (Figs 2, 6); (vi) forewing with subcostal crossvein present in basal section of wing (Fig. 1a–c); (vii) flagellomeres with five or six whorls of setae (Fig. 4c, d). However, as discussed later, the assignment of this species to Nothochrysinae is an “uncomfortable fit” (Makarkin and Archibald 2013: 125).

#### Classification – genus and species.

This species’ dark, mottled coloration, distinctive wing shape, and compressed venation make it highly recognizable among the Chrysopidae. Except in coloration (because of the fungal contamination in the holotype), the specimen reported here conforms completely to the generic description by Adams and Penny (1992b).

#### Redescription.

**Figure 3.**
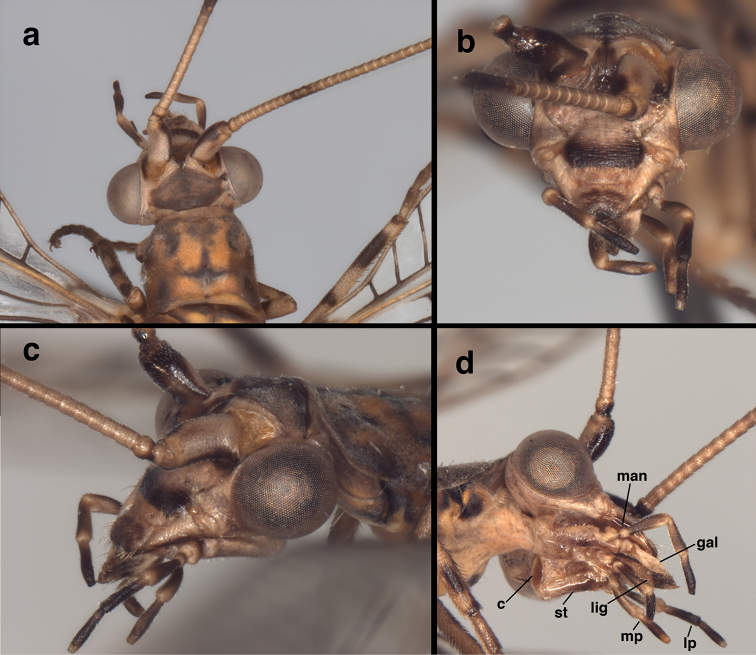
*Leptochrysaprisca* Adams & Penny (Peru, Amazonas, female, FSCA): Head and prothorax (**a**) head and prothorax, dorsal view (**b**) head, frontal view (**c**) head and anterior of prothorax, lateral view (**d**) head, posterolateral view. **c** cardo **gal** galea **lig** ligula **lp** labial palpus **man** mandible **mp** maxilary palpus **st** stipes.

**Figure 4.**
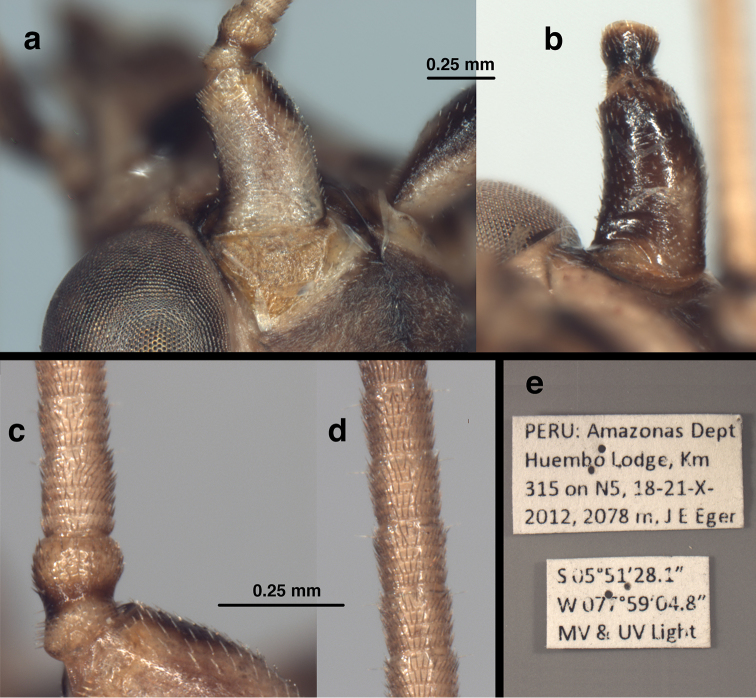
*Leptochrysaprisca* Adams & Penny (Peru, Amazonas, female, FSCA): Antenna and specimen labels (**a**) left scape, pedicel, dorsal torulus, tip of vertex, dorsal view (**b**) right scape, ventral view (**c**) pedicel, basal flagellar segments, dorsal view (**d**) distal flagellar segments, dorsal view (**e**) specimen labels. Scale between (**a**) and (**b**) applies to both (**a**) and (**b**); scale between (**c**) and (**d**) applies to both (**c**) and (**d**).

Head (Figs 3, 4). Width 2.1 mm (including eyes); ratio of head width to eye width = 2.8 : 1. Vertex slightly raised, round anteriorly, without prominent posterior fold, surface rugose, pilose, with setae small, pale. Distance between scapes 0.26 mm; distance between tentorial pits 0.65 mm; length of frontal region (midway between scapes to midway between tentorial pits) 0.56 mm. Frons rounded laterally, well delineated, extending caudally between antennae, appearing to terminate in an acute apex at anterior margin of vertex; interantennal surface sculptured longitudinally, with longitudinal crease mesally, small rounded ridges fanning out from between scapes below frontal toruli; anterior section with weak transverse striation. Gena (frontal view, Fig. 3b) appearing as rounded lobe from lateral base of scape to midsection of clypeus; tentorial pits on dorsal margin, near medial tip of lobe; insertion of mandibular base distinct, extending along full genal width. Clypeus relatively narrow basally, broader in center, narrowing distally; dorsal margin convex, lateral margins rounded, frontal margin straight; surface sculptured with transverse ridges except distally where ridges reduced, longitudinal. Frons, gena, base of clypeus rugose, without setae; distal part of clypeus, margins of mandibles with short to medium-length setae. Labrum narrower than clypeal margin, without ridges; dorsal surface somewhat sculptured; distal margin bilobed, with numerous long setae, especially on margin. Palpi elongate, tapered; venter of head with large, well-sclerotized cardo, stipes, narrow, elongate galea with conspicuous papilla; ligula elongate. Antenna length unknown (flagella broken); scape considerably longer than wide (length 0.57 mm, width 0.35 mm), with slight lateral bend; lateral margin slightly concave, mesal margin convex, surface with short setae throughout; pedicel length 0.23 mm, width 0.18 mm, with numerous short setae; flagellum with basal flagellomere distinct, somewhat elongate (length 0.18 mm, width 0.15 mm), midantennal flagellomeres about as long as broad (length 0.13–0.14 mm, width 0.13–0.14 mm); basal flagellomere with six whorls of brown setae extending distally, second to fifth flagellomeres each with four whorls of brown setae extending distally; sixth to distal flagellomeres each with five whorls of brown setae; all flagellomeres each with two elongate setae extending laterally from distal whorl. Flagellar setae in whorls stouter, longer than setae on vertex.

***Head coloration*** (Fig. 3). Antenna: dorsum, lateral sides of scape cream, with tan spot distolaterally; mesal, ventral sides dark brown; pedicel, cream to light brown with darker brown mesal band; flagellum cream to light brown, with eighth to twelfth segments dark brown; elongate setae, setae in whorls light brown to brown. Vertex dark brown; dorsal torulus golden tan; space between torulus and vertex cream; space between vertex and eyes, cranial area behind eyes cream to light brown. Frons mostly cream, with dark brown mark along lateral base of scape, below scape along frontal margin of torulus, across dorsum of frons, between scapes, extending in an acute angle to brown mark on vertex; frontal torulus golden brown. Tentorial pits, frons between pits cream; gena cream with light brown mark at base of eye. Exposed lateral surface of mandible dark brown basally, brown to light brown distally. Clypeus cream to light brown, with large, dark brown band across mesal section. Labrum with basal, lateral margins cream to light brown, central and distal areas dark brown. Exposed dorsal section of labium (ligula) cream basally becoming dark brown distally; ventral surface of labium mostly light brown. Maxilla (ventral) mostly light brown, with galea cream, cardo brown, stipes brown distally. Maxillary, labial palpi brown to dark brown, with intersegmental connections cream to light brown.

**Figure 5.**
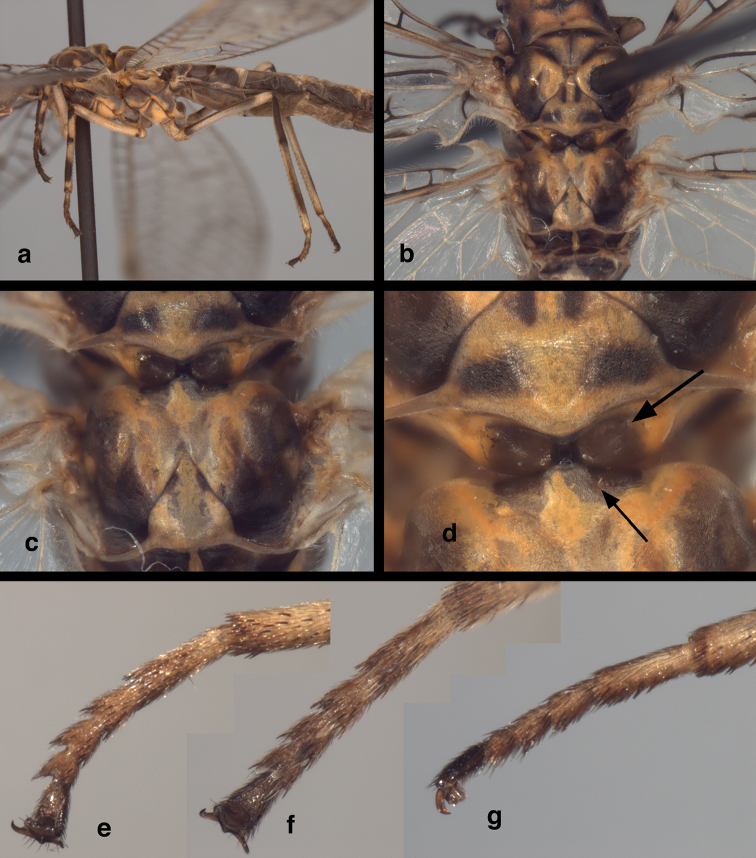
*Leptochrysaprisca* Adams & Penny (Peru, Amazonas, female, FSCA): Thorax (**a**) thorax and most of abdomen, lateral view (**b**) mesothorax and metathorax, dorsal view (**c**) mesoscutellum, metathorax, dorsal view (**d**) connection between mesoscutellum, metascutum (**e, f, g**) metatarsus, ventrolateral, ventral, lateral views, respectively. In (**d**), the lower arrow indicates the flat surface of the metascutum and its anterior quadrate protrusion; the upper arrow indicates the lobate lateral expansions of the mesoscutellum.

Thorax (Figs 3a, 5). Cervix brownish, appearing sclerotized or partially so. Dorsal thoracic surface mostly smooth, with waxy coating, golden orange, mottled with large brown markings, bearing fine, pale setae throughout. Pronotum large, appearing well sclerotized, with small transverse depression mesally, slightly broader than long (dorsal view), length 1.2 mm; width 1.5 mm; ratio of length to width = 0.8 : 1. Mesothorax, metathorax with surface shiny, smooth, appearing well sclerotized; each segment with dark brown markings. Mesopostscutum with bilobed enlargement on posterior margin; anterior margin of metascutum without raised process on anterodorsal margin, but with quadrangular knob on anterior margin extending forward toward bilobed enlargement of mesopostscutum. Legs elongate, slender, cream; each femur, tibia marked with two elongate brown marks, without prominent tibial spurs. Tarsus (ventrolateral side, all legs) with dense, robust, dark brown setae ventrally, more slender setae dorsally; terminus with pair of curved claws laterally, large pad mesally; claw amber, without basal enlargement, acute slender hook terminally.

**Figure 6.**
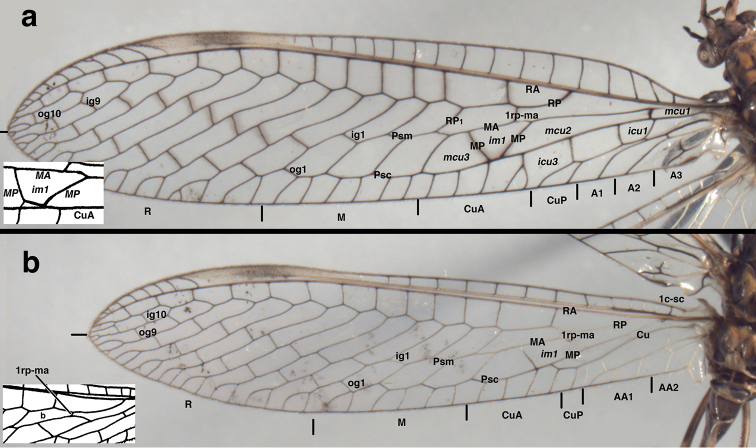
*Leptochrysaprisca* Adams & Penny (Peru, Amazonas, female, FSCA): Wings with selected features labeled (**a**) forewing, (**b**) hindwing. For comparison, the inserts depict the conditions on the *L.prisca* holotype for (**a**) the *im1* cell and (**b**) the proximal crossvein between RP and MA; images modified from Adams and Penny (1992b: fig 10). **A1, A2, A3** first, second, and third anal areas on wing margin, **1c-sc** first crossvein between the costa and subcosta **Cu** cubitus **CuA** anterior cubital area on wing margin **CuP** posterior cubital area on wing margin ***icu1, icu3*** first and third intracubital cells **ig, og** inner and outer gradate veins ***im1*** first intramedian cell **M** medial area on wing margin **MA, MP** anterior and posterior branches of the media ***mcu1, mcu2, mcu3*** first, second, and third medial cells **Psc** pseudocubitus **Psm** pseudomedia **R** radial area on wing margin **RA, RP** anterior and posterior branches of the radius **1rp-ma** first crossvein between RP and MA.

Wings (Figs 1, 2, 6). *Forewing*: Elongate, narrow, length 20.3 mm, maximum height 4.7 mm; ratio of length : maximum height = 4.3 : 1. Membrane transparent, uniformly covered with microtrichia. Trichosors (*sensu*Makarkin and Archibald 2013: 140–142) absent. Costal area narrow; tallest costal cell (#5) height 0.9 mm, 1.1 times width, 0.19 times height of wing; all costal crossveins simple, four c-sc crossveins before 1sc-r, fourteen c-sc after 1sc-r and before stigma, none within stigma. First sc-r crossvein (1sc-r) robust, angled basally; distal section of Sc extending into and fading within stigma, but not appearing to merge with C or RA; from no to two very faint sc-ra crossveins in stigma. Apical costal area (between C and RA) relatively broad apically; RA with ten to eleven anterior branchlets reaching apical region of wing margin. Furcation of R (R*f*) distal to 1sc-r crossvein, very much basal to furcation of M (M*f*). Radial area (between RA and RP) with single row of thirteen closed cells, only one ra-rp anterior to the first branch (RP_1_) stemming from RP; tallest cell (*rarp2*) height 0.9 mm, 0.9 times width; radial crossveins (ra-rp) straight. Radius with no crossveins to M before R*f*; 1rp-ma meeting MA at intramedian cell (*im1*). Media with one m-cu crossvein (therefore two medial cells, *mcu1*, *mcu2*) basal to *im1*. M*f* basal to first r-m crossvein (1rp-ma); angle of MA and MP broadly acute; MA becoming pale, diffuse and constricted between M*f* and insertion of 1rp-ma (both forewings) [holotype: MA thin in this area, but not pale]. Intramedian cell (*im1*) prominent, quadrangular in shape (but not *sensu*Breitkreuz et al. 2017: 29; see Discussion below), formed by MA anteriorly, MP basally, posteriorly, distally; anterior (MA) and posterior (MP) sides of *im1* roughly parallel for most of span; *im1* occupying entire vertical space between MA and CuA. MA and MP rejoining at anterodistal corner of *im1*, subsequently dividing at least once before meeting RP_1_, and probably a second time before meeting RP_2_. Third medial cell (*mcu3*) distal to *im1*, triangular. Two series of gradate veins diverging medially, converging distally. Nine inner gradates (in regular, sinuous series), extending beyond pseudomedia (Psm) in zigzag pattern across center of wing; ten outer gradates in slightly upturned series beyond pseudocubitus (Psc). Radial branches from RP elongate, wavy before inner gradates, less so after. Gradate cells rectangular. Approximately eight primary marginal forks reaching posterodistal margin (radial field) of wing. Cubital furcation (Cu*f*) near, but basal to m-cua crossvein. CuA with two simple crossveins to CuP, a distal third vein forked, reaching wing margin, and probably three additional simple branches reaching wing margin beyond forked vein. Second cubital furcation (CuP*f*) below *icu2*; thus, posterior wing margin with seven to ten cubital veinlets total (five to eight from CuA, two from CuP). A1, A3 simple, unforked; A2 forked before a1–a2 crossvein, with anterior branch reaching wing margin, posterior branch extending to A3, with short distal veinlet ending within cell (both wings). Jugal lobe large, usually folded beneath anal region, with jugal vein dark, extending to basal margin; basal margin with elongate, pale setae.

***Hindwing***: Length 17.5 mm, maximum height 4.0 mm. Costal base with well-developed frenulum bearing cluster of elongate terminal setae. Costal area narrow, with 15 crossveins before stigma, eight radial veinlets extending to C after stigma; no veins within stigma. Subcostal area without crossveins. M parallel and attached to R until just past 1c-sc; R*f* distal to 3c-sc. Radial area with single row of thirteen closed cells between RA and RP (= 13 ra-rp crossveins). Two series of gradate veins, roughly parallel, regularly spaced; nine or ten inner gradates extending beyond Psm; nine or ten outer gradates, regularly spaced, extending beyond Psc. Approximately eight primary marginal forks reaching posterodistal margin (radial field) of wing. Only one r-m crossvein (1rp-ma). First intramedian cell with MA as anterior margin, with MP as posterior margin basally, MP+CuA distally, distal arm either MP, MA_1_, or ma-mp crossvein). Cu sinuous, with two crossveins to A1, two branches reaching posterior wing margin before merging with MP. A1 with three veinlets reaching posterior wing margin; A2, A3 simple, unforked; one crossvein (a1–a2) between A1, A2; A3 forming base of jugal lobe; jugal lobe large, rounded.

***Coloration of forewing, hindwing*** (Figs 1, 2, 6): Membrane of both wings appearing clear, somewhat glossy. Stigma prominent, dark brown medially, golden on both ends. All veins dark brown except forewing with Sc golden, anterior base of MA yellowish; hindwing with 1c-sc, 3c-sc3 to 6c-sc, Sc beyond 6c-sc, base of R (including base of RA and RP), base of M, most of MA and its branches, base of Cu, all anal veins golden. Forewing with brownish suffusion around MP, inner gradates, and outer gradates.

**Figure 7.**
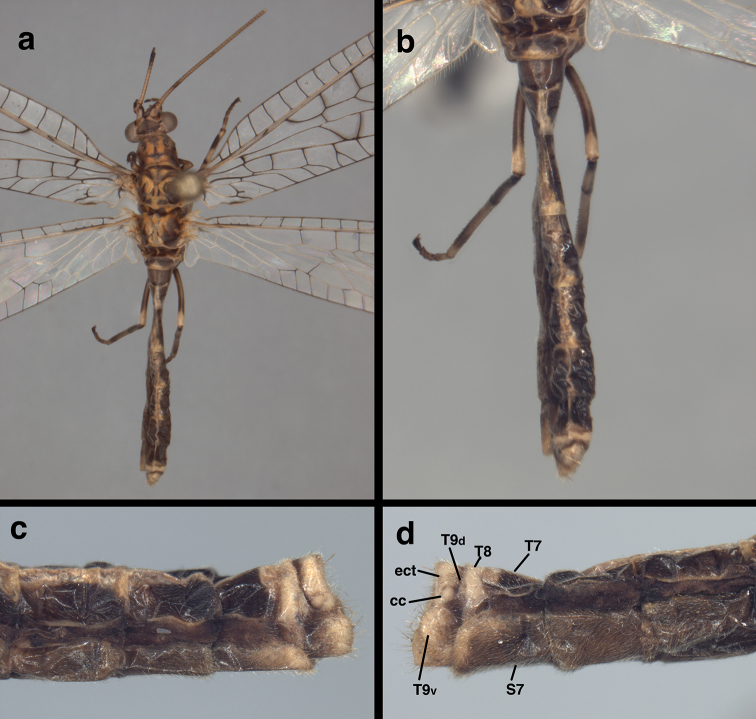
*Leptochrysaprisca* Adams & Penny (Peru, Amazonas, female, FSCA): Body and abdomen, external (**a**) body, dorsal view (**b**) abdomen, dorsal view (**c, d**) terminal abdominal segments, left and right, respectively, lateral views. **cc** callus cerci **ect** ectoproct **S7** seventh sternite **T7, T8** seventh, eighth tergites **T9d** dorsal section of large ninth tergite hidden beneath T8 **T9v** expanded ventral section of large ninth tergite encapsulating gonapophyses laterales.

**Figure 8.**
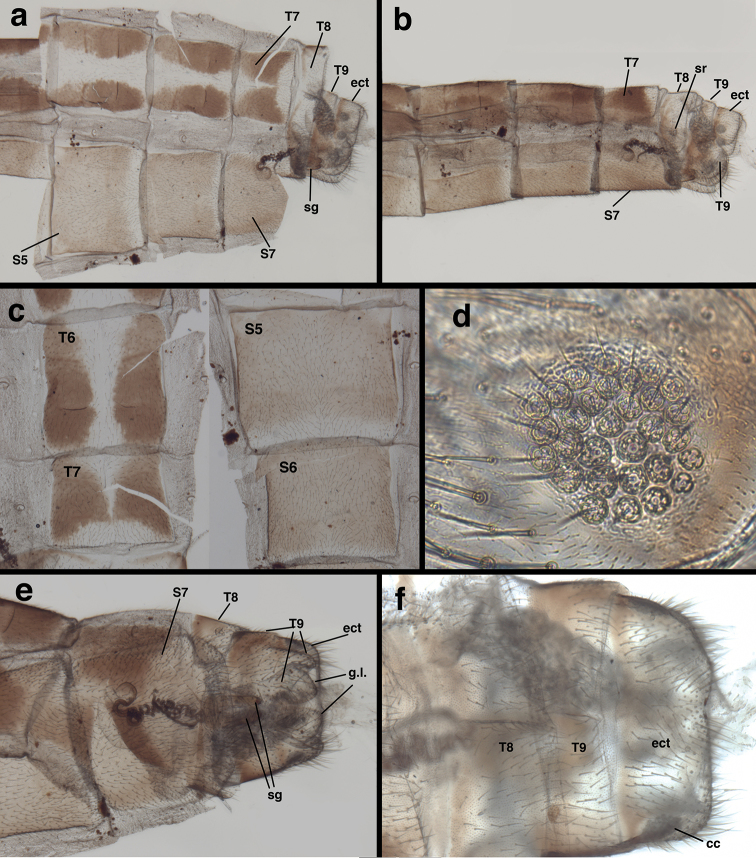
*Leptochrysaprisca* Adams & Penny (Peru, Amazonas, female, FSCA): Abdomen, cleared (**a**) abdominal integument dissected, segments A5-A7 with dorsal, lateral, and ventral surfaces in view, A8 with dorsal and lateral surfaces in view, A9, ectoproct in lateral view (**b**) segments A5-terminus, lateral view (**c**) abdominal integument, dorsal (T6-T7) and ventral (S5-S6) (**d**) callus cerci (**e**) terminal abdominal segments, ventral view (**f**) terminal abdominal segments, dorsal view. **cc** callus cerci **ect** ectoproct **g.l.** gonapophyses laterales **sg** subgenitale **sr** spiracle **S5, S6, S7** fifth, sixth, and seventh sternites **T6, T7, T8, T9** sixth, seventh, eighth, and ninth tergites.

**Figure 9.**
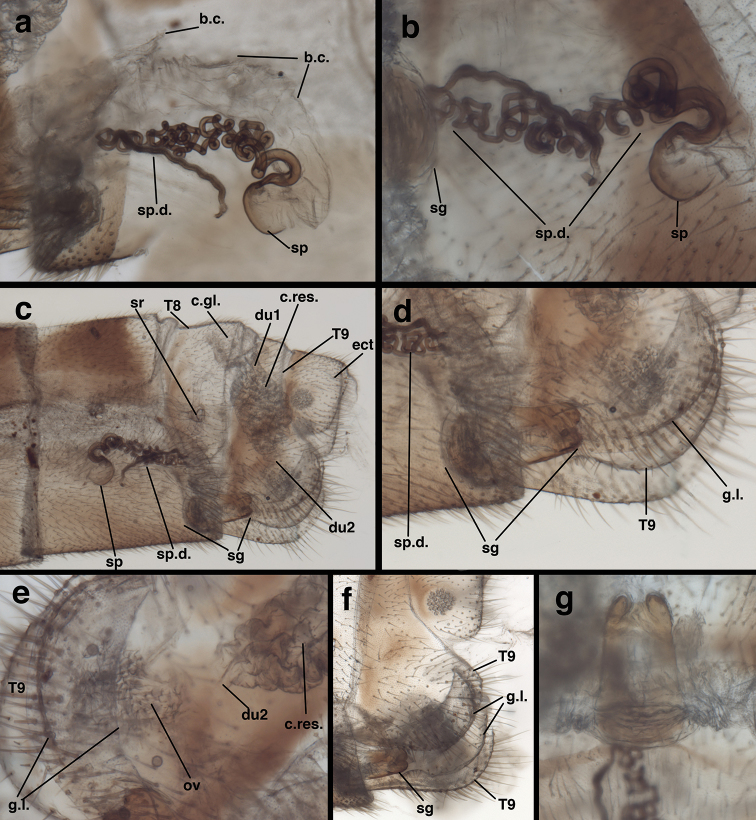
*Leptochrysaprisca* Adams & Penny (Peru, Amazonas, female, FSCA): Abdomen, genitalia, cleared (**a**) spermathecal complex (**b**) spermatheca and duct (**c**) terminalia, lateral view showing colleterial, spermathecal, and subgenitale complexes (**d**) subgenitale, lateroventral view (**e**) terminus, lateral view showing crescent-shaped gonapophysis lateralis (proximal and distal margins) encased between ventral extension of T9, spinose oviduct beneath (terminal end of duct 2 obscured) (**f**) gonapophyses laterales within extensions of T9, subgenitale beneath (**g**) subgenitale, dorsal view showing bilobed terminus. **b.c.** bursa copulatrix **c.gl**. colleterial gland (broken distally) **c.res.** colleterial reservoir **du1** large duct leading from colleterial gland to reservoir **du2** duct leading from colleterial reservoir to oviduct **ect** ectoproct **g.l.** gonapophysis lateralis nestled beneath T9 **ov** oviduct **sg** subgenitale **sp** spermatheca **sp.d.** spermathecal duct **sr** spiracle **T8, T9** eighth and ninth tergites.

Abdomen (female, Figs 7–9; male unknown). Tergites, sternites, pleural region covered with relatively dense setae of uniformly short length; microsetae present, no microtholi. T6 (lateral view) length 1.2 mm, ~2.1× height, approximately same proportions as T7 (length 0.9 mm, ~1.7× height). S6 length 1.2 mm, ~2.0× height; S7 length 1.2 mm, approximately 2.0× height. Tergites roughly rectangular, with edges acute or slightly rounded, ventral margins straight; lateral (dark brown) regions more rigid and robust than mesal section. Sternites quadrate, uniformly colored and rigid throughout. Spiracles located in pleural membrane, slightly closer to sternites than to tergites, roughly oval externally, not enlarged; atria slightly enlarged, rounded, with single tracheae. Coloration mostly dark brown with cream stripe on dorsal midline; distal tip of T7, T8, posterior region of T9, ectoproct, gonapophysis lateralis cream; setae, setal bases pale; callus cerci cream.

***Female terminalia*** (Figs 7c, 7d, 8, 9c–f): Callus cerci (Fig. 8d) approximately circular, diameter 0.14-0.16 mm, with ~30 trichobothria of mixed length. Tergite 8 much narrower than T7 (lateral view), much taller than T7, extending well beyond distal margin of S7, with rounded ventral margins, bearing spiracle in lower sclerotized section. Tergite 9, ectoproct distinctly separate; T9 with dorsal margin narrow, less than half length of T7, becoming broad, bulbous ventrally, completely encasing gonapophyses laterales, ventral margin rounded, over three times length of dorsal margin. Sternite 7 roughly quadrate, with dorsal margin straight, approximately same height as S6, rounded and sloping abruptly in distal quarter, base at ventral margin extending distally as apical ledge, without knob; posteroventral setae slightly longer, more dense than other setae. Ectoproct with dorsal margin twice as long as dorsal margin of T9, ventral margin rounded, tucked below distal bulge of T9, bearing callus cerci near posterior margin; callus cerci about half width of segment. Gonapophysis lateralis well sclerotized, broadly crescent shaped, mostly enclosed by distal extension of T9 (Fig. 9f), with sparse distribution of small setae on distal, exposed surface only, not on basal surface.

***Colleterialcomplex* (*posterior to anterior*)** (Fig. 9c, e): Oviduct immediately behind gonapophyses laterales, chamber setose (setae arising from large bulbous bases); no transverse sclerification found. Two sets of glands entering oviduct: posterior gland, fluted, with rough, setose surface, thin duct bearing secondary gland or small reservoir before opening to oviduct; anterior gland (probably the primary colleterial gland) distally with globate colleterial reservoir larger than width of T9, heavily textured surface with numerous rounded folds and some setae, entering oviduct via short, somewhat broad duct. Colleterial gland (anterior end missing) entering colleterial reservoir between T8 and T9 via broad, robust, membranous duct, gland probably large, with broad, structured, circular base, setose membranous sides, at least distally.

***Bursalcomplex*** (Fig. 9a): Bursa copulatrix with two sections; larger section consisting of delicate, transparent membrane with transverse, angled folds, covering entire spermatheca and spermathecal duct; smaller section leathery, triangular, attached above membranous section; two sections fusing distally before entering chamber above subgenitale; bursal glands not found. Spermatheca bowl-shaped, somewhat transparent, invaginated, apparently open to large section of bursa via slit on basal (proximal) side of bowl and perhaps on spermathecal duct. Spermathecal duct well sclerotized, very long, with coiled section extending about 0.75 length of S7, straight section doubling back almost completely; basal section, tightly coiled, curved on itself, with smooth surface; distal section mostly straight, with some slight bending, dense, surface with brushy covering of setae; region between two sections in contact with subgenitale complex.

***Subgenitale*** (Fig. 9d, g): Basal section well sclerotized, extending from leathery, partially sclerotized, membranous base; distal section elongate, rounded, robust laterally, flat mesally, protruding distally between gonapophyses laterales, well beyond S7, with patch of approximately ten robust setae ventrally, shallow bilobed tip distally. Basal membranous section considerably shorter than sclerotized distal section, extending from sturdy membranous fold within ventral tip of S7, rounded proximally, folded throughout.

#### Biology.

**Figure 10.**
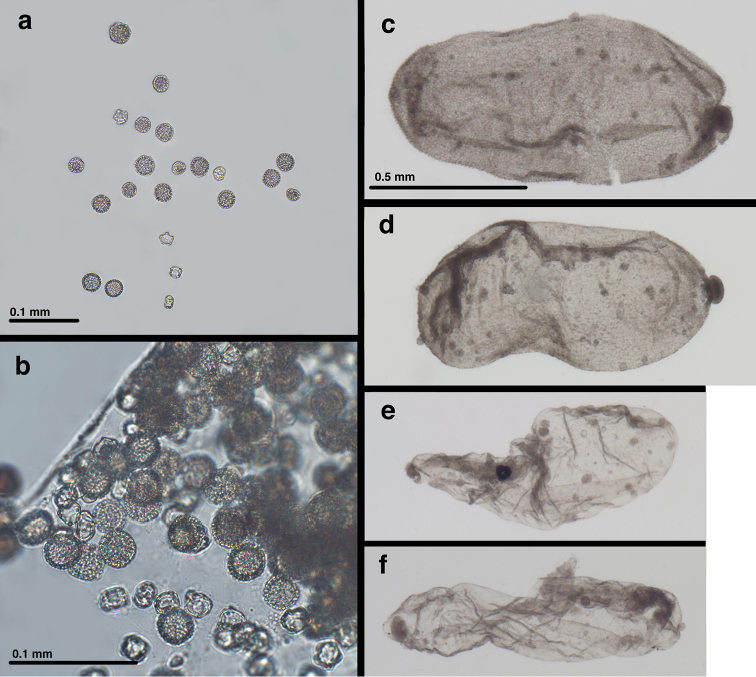
Contents of *Leptochrysaprisca* Adams & Penny abdomen (after clearing with KOH) (Peru, Amazonas, female, FSCA): Pollen and parasitoids. (**a, b**) pollen from gut (**c, d**) two of five robust parasitoid larvae from abdominal cavity (**e, f**) probably exuviae (two of five) from previous parasitoid instar. Scale on (**c**) applies to (**c, d, e, f**).

***Abdominal contents – pollen***: A label on the type specimen indicated that it was taken from *Baccharislatifolia* (Ruiz & Pav.) Pers., a flowering shrub that is common throughout much of South America, including Peru. Adams and Penny (1992b) noted pollen in the gut contents. Thus, it is not surprising that both the foregut and hindgut of the female specimen studied here also were filled with pollen. The pollen grains were of several sizes and shapes: predominately large and round, but also small and round, as well as small and quadrate (Fig. 10a, b).

***Abdominal contents – parasitoids***: After the abdomen was cleared, it was found to contain a number of parasitoid larvae (probably Hymenoptera). The parasitoids were also cleared during the process, and the resulting specimens consisted of two types. First, there were five robust larvae with a textured, scabriculous integument throughout, a rounded knob at one end, and a pair of small protrusions at the other end. The interior of these specimens appeared empty (Fig. 10c, d)); no mouthparts or other structures were visible. Second, there are five smaller, more delicate specimens, consisting of clear, smooth integuments without structures or setae (Fig. 10e, f). It is possible that these clear specimens are the exuviae of previous instars or a very different form or stage of parasitoid than the robust ones. It is noteworthy that the abdomen of the lacewing host was relatively slender for a well-sclerotized female (Fig. 7).

***Larvae***: Discovery of *L.prisca* larvae would greatly help to decipher the phylogenetic relationships of the genus *Leptochrysa*. Unfortunately, the larvae of this genus remain unknown. Descriptions are available for comparison with the larvae of several Nothochrysinae genera: *Kimochrysa* Tjeder, *Pimachrysa* Adams, *Dictyochrysa* Esben-Petersen, *Hypochrysa* Hagen (one species each), and several species of *Nothochrysa* (see review by Tauber et al. 2014). The known *Nothochrysa* larvae are debris-carriers, whereas the known larvae of other genera are naked.

#### Known distribution.

Thus far, there are only two records for this species, and both are from the Amazonas region of northern Peru. The specimen studied here is from Huembo Lodge, a reserve run by the Ecoan Andean Ecosystems Organization, and located on Hwy 5N southwest of Pomacochas.

#### Intraspecific variation.

Other than the discoloration and damage caused by fungal growth on the holotype and some variation in the number of gradate cells, the two known *L.prisca* specimens show significant similarity. However, there is one notable area of variation in the forewing – the posterior margin of the intramedian (*im1*) cell. This variation, although subtle, proves to be useful in deciphering the venation of the *Leptochrysa* forewing.

In both specimens of *L.prisca*, the MP forms the posterior margin of the *im1* cell, and MP meets CuA at the posterobasal corner of *im1*. In the left and right wings of the holotype, MP and CuA clearly remain distinct (see insert on Fig. 6a). When the MP reaches the CuA, the two veins do not fuse; they extend distally along separate trajectories. MP alone forms the posterior (lower) border of *im1*. CuA runs below MP and forms the base of a narrow triangular cell beneath the *im1*. A very short 2mp-cua crossvein or a branch of the MP extends anteriorly from the posterodistal corner of *im1*, and CuA continues distally.

In comparison, on the second specimen (left and right wings), MP and CuA appear as a single vein along the full posterior span of the *im1* cell. However, on the basis of the holotype’s venation, I assume that the two veins remain juxtaposed, but separate along this span.

The configuration described above appears to be unusual within Nothochrysinae. Although variable, the *im1* in Nothochrysinae generally is triangular, being formed by MA, MP, and crossvein 1ma-mp. (Note: Breitkreuz et al. 2017 would consider this configuration as "pseudotriangular", but see reasons presented by Tauber (2019) for identifying this configuration as "triangular".). In contrast, the findings here indicate that the *im1* is composed entirely of the anterior and posterior branches of M, without a crossvein. Thus, the *im1* cell of *L.prisca* more closely aligns with a category of *im1* cells that is not usually reported for Nothochrysinae: shaped like a triangle or quadrangle, but without a crossvein forming a portion of the cell. Thus, the cell’s quadrate shape and configuration in *L.prisca* elicit questions concerning the identity of the “crossvein” proposed to close the distal ends of the *im1* within other genera of Nothochrysinae. It is also noteworthy that on both the holotype and the second specimen of *L.prisca*, it is not clear whether the MP furcates at the posterodistal corner of the *im1*. If it does, then the CuA vein extending from the posterodistal corner of the *im1* would be a fused CuA+MP and thus part of the Psc.

## Discussion

*Leptochrysaprisca* lacks several characteristics that are typically found in Nothochrysinae, and it also expresses some features that are unique among chrysopids or characteristic of ancient chrysopid subfamilies. As a result, the placement of the genus in Nothochrysinae remains unsettled (Makarkin and Archibald 2013). Below I discuss some of the features of interest.

*Relative lengths of Sc and RA of forewing*: In the elongated wings of *L.prisca* (both specimens), the Sc extends only partially into the stigma where it appears to dissipate, and the RA, which reaches well beyond the stigma almost to the apex of the wing, has numerous distinct veinlets that extend to the costa. According to Adams and Penny (1992a), the elongation of the wing and such a configuration are typical of species in two genera of Mesochrysopidae. And, according to Makarkin and Archibald (2013: 125), the *L.prisca* configuration of Sc and RA resembles that of the genus *Protochrysa* Willmann & Brooks in Limaiinae (Chrysopidae). In addition, aspects of the arrangement, e.g., veinlets on the posterior margin of the wings and perhaps the separation of the R and Sc near the tip of the wing, are typical of some Apochrysinae (see Winterton and Brooks 2002). In any case, none of the above features are known from other Nothochrysinae.

*First intramedian cell of forewing*: (i) Relationship of CuA to *im1*. In *L.prisca*, the MP runs parallel with the CuA, either in contact with it or very closely nearby, to form the posterior margin of the *im1.* Among the Nothochrysinae, such a close association between the *im1* and CuA is shared only with *Triplochrysapallida* Kimmins. In this species, the *im1* cell is bounded posteriorly by MP and CuA (see New 1980: fig. 13); however, it is unknown if the two veins run separately and in parallel as they apparently do in the *L.prisca* studied here.

(ii) Vertical space between MP and CuA. In most Nothochrysinae, the *im1* occupies about one-half to two-thirds the vertical distance between MP and CuA (see New 1980, Brooks and Barnard 1990). In *L.prisca*, the *im1* cell, which is quadrate in shape, occupies the entire vertical space between MP and CuA. Among the Nothochrysinae, an *im1* that fills the entire vertical space between MP and CuA is shared only with *T.pallida* (see New 1980: fig. 13).

(iii) Modification of the second m-cu crossvein. As described above, the *L.prisca* holotype has a small second m-cu crossvein (mp-cua), and the second specimen entirely lacks a second m-cu crossvein. This reduction/loss appears to be unique among Nothochrysinae (see Adams 1967, Brooks and Barnard 1990, Archibald and Makarkin 2015: 361). Makarkin and Archibald (2013) consider this feature, as well as the basal tapering of the *im1* in *L.prisca*, to be suggestive of Limaiinae.

*Rectangulargradatecells*: Adams and Penny (1992a) noted that the gradate cells of *L.prisca* are clearly quadrangular in shape and very unlike the polygonical gradate cells typical of modern chrysopids. They considered such cells to represent a plesiomorphic condition among chrysopoids because even some mesochrysopids (e.g., *Mesypochrysa* Martynov) have gradate cells that are more typical of modern chrysopids. As they pointed out, the critical question is whether these cells are truly plesiomorphic or secondarily derived, perhaps associated with wing elongation.

*White “break” in MA of forewing*: The mostly dark wing venation of *L.prisca* accentuates a characteristic that is widespread among Nothochrysinae males and females, but apparently has been unreported. The media (MA), directly below or near the insertion of the rp-ma crossvein, is interrupted by a short span that is white and either broken or diffuse. The span contains what appears to be a tracheal branch. Distal to the white span the MA reassumes its normally defined, dark structure. These features are readily noticeable on the *L.prisca* specimen described here, less so on the holotype which has generally discolored (dark) venation. However, the holotype shows the narrowing of MA in the region.

The white “break” or narrowing of MA observed in *L.prisca* also occurs in all New World genera of Nothochrysinae: *Asthenochrysa* Adams & Penny, *Nothochrysa* McLachlan, *Pimachrysa* Adams (at least four species: *P.albicostalis* Penny, *P.fusca* Adams, *P.intermedia* Adams, and *P.nigra* Adams), and, as well as in *Dictyochrysapeterseni* Kimmins, *Hypochrysaelegans* (Burmeister), *Nothochrysasinic*a Yang, and perhaps other species in the Old World. The feature is readily seen in *Nothochrysacalifornica* Banks and *Pimachrysa* species because they, like *L.prisca*, have mostly dark veins. In *Asthenochrysa* and the South American species *Nothochrysaehrenbergi* Tauber, which have pale or mottled wing venation, the white span in the MA can be difficult to discern, but it is present. This character has not been reported for other chrysopids; its phylogenetic importance, if any, is unknown.

*Proximal crossvein between RA and MA of hindwing*: Adams and Penny (1992a, b) and Makarkin and Archibald (2013) reported that *Leptochrysa* is the only extant chrysopid genus in which the hindwing has a basal crossvein connecting RP and MA (rp-ma, identified and illustrated as the basal vein of the “b cell” by Adams and Penny; see insert on Fig. 6b). And, according to both sets of authors, this feature occurs in most Limaiinae and only in some early Eocene Nothochrysinae, perhaps most notably *Archaeochrysa* Adams. It was not reported from extant Nothochrysinae. However, some exceptions may have been overlooked: *Pimachrysanigra* (Adams), *Kimachrysaafricana* (Kimmins), and *Nothochrysaturcica* Kovanci & Canbulat, all of which are modern species in Nothochrysinae (Tjeder 1966, Adams 1967, Tauber 2019), and all of which appear to have an rp-ma crossvein. Thus, the value of the character as an indicator of phylogenetic position may not be as strong as originally thought.

*Surface of the wings*: *Leptochrysaprisca* is the only chrysopid known to have the membranous surfaces of its forewings and hindwings covered with microtrichia. As indicated by Adams and Penny (1992a, b), such an extensive covering of microtrichia occurs in Hemerobiidae and other Neuroptera but is not known in other chrysopids. Rather, in Chrysopidae, microtrichia are restricted to the bases of the forewings, and do not occur on other surfaces of the wings.

*Metanotal expanded (raised) knob*: A recent study by Breitkreuz (2018) reported that the dorsal surface of the anterior metascutum in Nothochrysinae typically has a raised expansion or knob. I have confirmed this feature for all New World genera and several Old World genera of Nothochrysinae. However, on the holotype and the specimen of *L.prisca* described here, the dorsal surface of the metascutum has no knob-like protrusion. In contrast, the anterior margin of the *L.prisca* metascutum is enlarged anteriorly as a flat, rectangular protrusion (Fig. 5c, d). Similarly, the metascutum of the holotype is depressed, and the anterior margin is slightly protruding and quadrate. This type of anterior extension/protrusion is only known for *L.prisca*, not for other Nothochrysinae. It is unknown whether this feature is independent or an alternate, homologous expression of the metascutal expansion/knob.

It should be noted that the paired, lobate structures on the posterior margin of the *L.prisca* mesoscutellum (where it meets the metanotum, Fig. 5c, d) are larger and more mesally located than those reported from *Nothochrysa* (cf., Tauber 2019, fig. 4b). Perhaps, the enlargement and placement of the above mesonotal and metanotal structures are associated with flight involving narrow wings.

*Sclerotization of thoracic and abdominal tergites*: Relative to other Nothochrysinae I have studied (mainly New World species), *L.prisca* seems to have an unusually well-sclerotized thorax (i.e., it has a rigid form and is sturdy). In other New World Nothochrysines, the thoracic integument is soft and flexible, and it often appears collapsed in pinned specimens. The feature is especially apparent in *Asthenochrysa*, but I do not know whether it occurs among Old World Nothochrysines.

Also, the presence of paired tergites and the pronounced softness along the dorsal abdominal midline of *L.prisca* (Fig. 8a, c) are unique, as far as I know. Chrysopid abdominal tergites are generally well sclerotized and rigid mesally and less so laterally. Both of the above features are in need of comparative study.

## Conclusion

The phylogeny of *Leptochrysa* remains enigmatic, and the assignment of the genus to Nothochrysinae (indeed, to Chrysopidae) is unsettled. Resolution of its phylogenetic relationships within Neuroptera awaits studies of the larval stages and molecular analyses.

## Supplementary Material

XML Treatment for Leptochrysa prisca
